# Computational Approach to Seasonal Changes of Living Leaves

**DOI:** 10.1155/2013/619385

**Published:** 2013-02-25

**Authors:** Ying Tang, Dong-Yan Wu, Jing Fan

**Affiliations:** ^1^School of Computer Science and Technology, Zhejiang University of Science and Technology, Hangzhou 310023, China; ^2^Key Laboratory of Visual Media Intelligent Processing Technology of Zhejiang Province, Hangzhou 310023, China

## Abstract

This paper proposes a computational approach to seasonal changes of living leaves by combining the geometric deformations and textural color changes. The geometric model of a leaf is generated by triangulating the scanned image of a leaf using an optimized mesh. The triangular mesh of the leaf is deformed by the improved mass-spring model, while the deformation is controlled by setting different mass values for the vertices on the leaf model. In order to adaptively control the deformation of different regions in the leaf, the mass values of vertices are set to be in proportion to the pixels' intensities of the corresponding user-specified grayscale mask map. The geometric deformations as well as the textural color changes of a leaf are used to simulate the seasonal changing process of leaves based on Markov chain model with different environmental parameters including temperature, humidness, and time. Experimental results show that the method successfully simulates the seasonal changes of leaves.

## 1. Introduction

The seasonal changes of trees vary the appearances of trees through seasons, which include shapes and textures of the leaves, flowers, and fruits. Among these, the change of leaves constitutes the most important part of the seasonal changes of trees. In this paper, we focus on how to compute the leaf changing during different seasons.

As we observe the changes of leaves from spring to winter, most leaves become withered and curled up due to the influences of environmental factors [1]. Besides, the leaves usually turn from green to yellow during the aging process and finally fall off to the ground. According to the above observation, the seasonal changes of leaves are simulated in terms of their geometric deformations as well as their textural colors transitions. There is a lot of research work done in simulating 3D shape changes of leaves the occurring during the withering process of leaves. Most of these methods generate the 3D deformation of leaves based on the changes of veins [2–7]. For veins-driven methods [3, 4, 6, 7], each vertex in the 3D model of a leaf is deformed to the nearest vertex in the interactively generated veins and deformations are controlled by dragging some vertices in the veins. These methods involve, much user interaction to extract the skeleton of the leaf, and the generated results are not realistic enough. The method proposed by Chi et al. [8] combines the veins with a double-layered model of the leaf and simulates the deformation process more realistically. However, this method is computationally intensive and difficult to implement due to the complex computation. In this paper, we propose a new improved method using mass-spring model and grayscale mask map to simulate the deformation process of leaves with simplified computations and realistic results.

In order to simulate textural colors of leaves, the Phong lighting model with a diffuse component derived from leaf pigments is adopted to directly compute the reflections on the surfaces of leaves [9]. Other methods use the technique of texture mapping to produce the leaves' appearances, and the textures can be changed to reflect the appearance changes of leaves [10]. In our method, we apply multiple textures to represent appearance changing of leaves in different seasons.

In order to efficiently simulate the seasonal changes of leaves, we combine the changes of geometric shape and textural color of the above methods in our algorithm to produce the results. The Markov chain model is used to show the state transfer of leaves in the dynamic growing process of trees. The following sections are arranged as follows. In Section 2, the related work is introduced. We describe the modeling of three-dimensional leaves in Section 3.  Section 4 focuses on the implementation of geometrical changes of leaves based on improved mass-spring model. In Section 5, the Markov chain-based method is described to compute different states of leaves combining the texture and geometry changes. We show our experimental results in Section 6 and conclusion in Section 7.

## 2. Related Work

The work related to the simulation of seasonal changes of leaves includes leaf modeling, leaf deformation, and leaf appearances rendering. For leaf modeling, there are L-system-based and image-based methods. The L-system-based methods model leaves with self-similarity [11, 12]. As for image-based modeling methods [13, 14], usually the feature points on the edge of the leaf are extracted from the scanned leaf image, and the geometric shape of the leaf is represented by the triangular meshes produced by Delaunay algorithm [15]. According to the botanical characteristics of the leaf, Dengler and Kang claim that leaf shapes have a close relationship with leaf veins [16] which is used to generate the shapes of leaves. Runions et al. present the biologically motivated method to construct leaf veins with user interaction [17]. Besides user interaction, the leaf veins are generated by fixing the start points and setting the control points of veins according to the sum of a fixed value and a random parameter between zero and ten [18]. Chi et al. [8] introduce an improved method to construct the leaf vein skeleton which generates the main vein and the branch vein separately, and the leaf model is built by a double-layered mass-spring model. These methods produce the relatively complex leaf models which reflect the characteristics of leaf's geometric shapes. In this paper, we generate the optimized triangular mesh to represent the leaf model by two steps. In the first step, the key points on the edge of the leaf are obtained through user interaction. Then, the optimized leaf triangular mesh is generated by improved Delaunay algorithm in the second step. Instead of generating the leaf veins explicitly in the modeling procedure, we emphasize leaf veins with a user-specified mask in the process of leaf deformation.

The leaves gradually become withered and curled up during the transitions of different seasons. The deformation of geometric shapes of leaves is very important to simulate the seasonal changes. The 3D deformation algorithms are mainly classified into two categories, which are free-form-based deformation methods [19] and physically based deformation methods [20]. Free-form-based deformation methods are widely used in the field of computer animation and geometric modeling [21]. These kinds of methods embed the objects into a local coordinate space and transform the local space to make the objects deformed. There are two common physically based deformation methods: skeleton-based method and mass-spring-based method. The deformation method based on skeleton is relatively simple [7] and produces more realistic deformation results of leaves. However, it requires much human interaction. Mass-spring model is more frequently used in fabric deformation [22]. Tang and Yang [23] adopt the mass-spring model to generate the deformation of leaves, in which the mesh of the leaf is not optimized, and the deformation effects are relatively unnatural and difficult to control. Double mass-spring model proposed by Chi et al. [8] is capable of simulating the changes of leaves more realistically. However, it is complex and difficult to be implemented.

In order to simulate color changes of leaf surfaces in various environmental conditions, Phong lighting model considering leaf's pigments [9] and the technique of texture mapping [24] have been adopted. The texture images of leaves can be obtained by scanning real leaves [25] or texture synthesis [26]. Desbenoit et al. [10] applies open Markov chain model to decide which texture images are mapped to certain leaves to simulate the aging process of the leaves. In this paper, we also adopt the Markov chain model to statistically determine the distribution of leaves, textures on the tree under the influence of environmental factors including temperature and humidness. 

## 3. Modeling Three-Dimensional Leaves 

In this paper, we apply the image-based approach to model the geometric shapes of three-dimensional leaves [27, 28]. First, the key points on the edge of the leaf are obtained through user interaction, and then the triangular mesh of the leaf is constructed by Delaunay triangulation through incremental insertion of points [29, 30]. Finally, the optimization procedure is employed to compute the high quality mesh with even-sized triangles.

**Figure 1:**
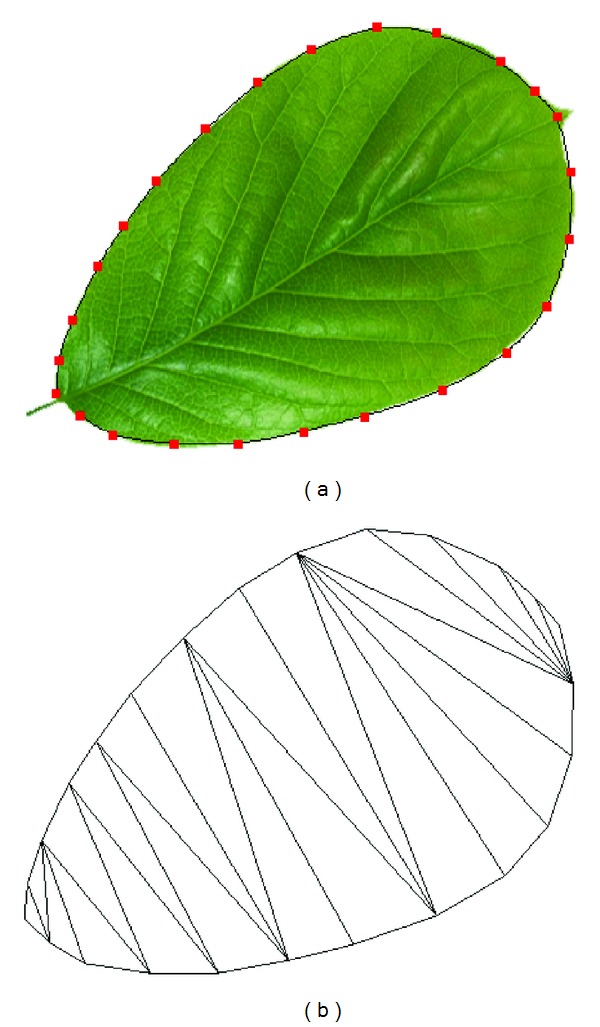
(a) The B-spline curve with key points selected by the user; (b) the Delaunay triangulated mesh of the leaf.

Instead of adopting the automatic edge detection methods to extract the leaf contour, we provide the interface to make the user interactively select the edge points of the leaf. After the selection of edge points, the smooth B-spline curve running through these points is automatically generated to approximate the leaf edges [31]. The B-spline edge which passes through the user-selected points is shown in Figure 1(a), from which we find that the curve represents the real leaf edge well. If more control points are selected, the edge is more accurate. The generated B-spline curve is sampled to get the key points which are to be used in Delaunay triangulation.

The Delaunay triangulation method is usually used to generate a triangulated irregular network (TIN) [32]. The Delaunay triangles are a set of connected but not overlapping triangles, and the circumscribed circle of the triangles does not contain any other point in the same region. Unfortunately, the initially triangular mesh generated with key points on the edge usually contains some long and narrow triangles, as shown in Figure 1(b). The leaf mesh with such bad quality triangles would make the leaf deformation unnatural. Instead, we need to generate a high quality leaf mesh with even-sized triangles. So we optimize the triangular mesh based on the subdivision method in [33]. An even-sized triangular mesh is obtained by repeating the following two steps: (1) relocate the vertex position; (2) modify the connection properties of triangles.

**Figure 2:**
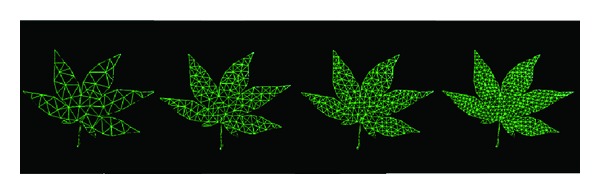
Triangular meshes of the maple leaf produced by a different number of iterations.

The high-resolution triangular mesh produces more natural and smooth deformations. However, more triangles in the mesh would lead to more time to compute the deformation. According to the triangulation algorithm, the subdivision level of triangular mesh is related to the number of iterations. Usually, we set the number of iterations to be 160 in our implementation, which is enough to produce the subdivided triangular mesh capable of natural deformation within acceptable time. In Figure 2, we show the triangular mesh models of the maple leaf produced by a different number of iterations.

## 4. Deformations of Leaves Based on Improved Mass-Spring Model

Leaves become slowly curled up as the season changes. This phenomenon is mainly caused by the different structures of the upper and bottom surfaces of a leaf, which have different amounts of contraction during the dehydration process. To take into account the differences between the upper and bottom internal structures of a leaf, we introduce the improved mass-spring model to make leaf deformation more realistic.

### 4.1. Numerical Calculation and Constraints

The mass-spring model is widely used in the simulation of the deformation of soft fabrics [34]. This model consists of two important parts: a series of virtual particles and the corresponding light springs of natural length nonequal to zero. The deformation of the object is determined by the displacements of particles after they are stressed. The springs connecting the particles constrain the movement of particles. The triangular mesh model of a leaf can be used as the mass-spring model, where the mesh vertices are regarded as particles and the edges are as springs [8].

There are internal and external forces acting on the springs, and we denote the joined forces as *F*_*i*,*j*_(*t*). The force distribution is computed by Newton's laws of motion, and explicit Euler's method is adopted to find the numerical solution of the model. The equations to compute the acceleration, particle velocity, and particle displacement are listed as follows:

(1) $$\begin{array}{c} a_{i,j}\left(t+\Delta t\right)=\frac{1}{\mu_{i,j}}F_{i,j}\left(t\right),\\ v_{i,j}\left(t+\Delta t\right)=v_{i,j}\left(t\right)+\Delta t\cdot a_{i,j}\left(t+\Delta t\right),\\ P_{i,j}\left(t+\Delta t\right)=P_{i,j}\left(t\right)+\Delta t\cdot v_{i,j}\left(t+\Delta t\right). \end{array}$$

In the above equations, the mass of a particle is denoted as *μ*_*i*,*j*_, the acceleration is denoted as *a*_*i*,*j*_, the velocity of a particle is denoted as *v*_*i*,*j*_, and the particle's displacement is denoted as *P*_*i*,*j*_. The time step is denoted as Δ*t*, the value of which is important in computing the desirable deformation. The time step needs to be small enough to ensure the stability of the numerical calculation. Otherwise, dramatic changes of particle positions would be incurred by large time step values.

Actually, the deformation curve of a leaf under forces is not ideally linear. If we directly compute the deformation with the above equations, the problem of “over elasticity” would occur, that is, the deformation of the springs would exceed 100%. To overcome this problem, we adopt the method of constraining velocities to constrain the deformation of the springs [35]. The basic idea is as follows. Particle *u* and particle *v* are the ends of spring *s*.*V*_*μ*_(*t*) and *V*_*v*_(*t*), respectively, represent the velocity of particle *u* and particle *v* at time *t*. Assume that the relative velocity between the two particles is *V*_*μ*,*v*_(*t*), and the relative position is *P*_*μ*,*v*_(*t*), the new relative position after one time step *P*_*u*,*v*_(*t* + Δ*t*) is computed by constraining the velocity of the particle. If *P*_*u*,*v*_(*t* + Δ*t*) satisfies (2), the velocity is updated [35]. Otherwise, it is not updated

(2) $$\begin{array}{c} P_{u,v}\left(t+\Delta t\right)=\left|P_{u,v}\left(t\right)+V_{u,v}\left(t+\Delta t\right)\cdot \Delta t\right|\leq \left(1+\tau_{c}\right)\cdot L. \end{array}$$

In (2), *L* presents the natural length of the spring without any forces exerted, and *τ*_*C*_ is the threshold of deformation. This equation guarantees that when the value of *τ*_*c*_ is set to be 0.1, the maximum deformation length of the spring does not exceed 10 percent of the natural length. In other words, the difference between *P*_*u*,*v*_(*t* + Δ*t*) and *P*_*u*,*v*_(*t*) should be within 10 percent of the natural length.

### 4.2. Deformation

The key of shape deformation is to compute the changes of the position of each particle. If each particle has the same mass value, the relative displacements in directions *x*, *y*, and *z* only depend on the joint force in each direction. For a relatively high-resolution mesh model with nearly even-sized triangles, the joint forces between most particles and its adjacent particles would not differ enough to make desirable deformations. Thus, the uniform mass of all particles is not in favor of generating the nonuniform deformation results relative to different leaf regions, for example, the regions near edges usually undergo more deformation than the center regions. To enhance the change of the relative displacement of each particle and generate the adaptively deformed results for different leaf regions, we adaptively allocate the mass values to different particles in our improved deformation model.

**Figure 3:**
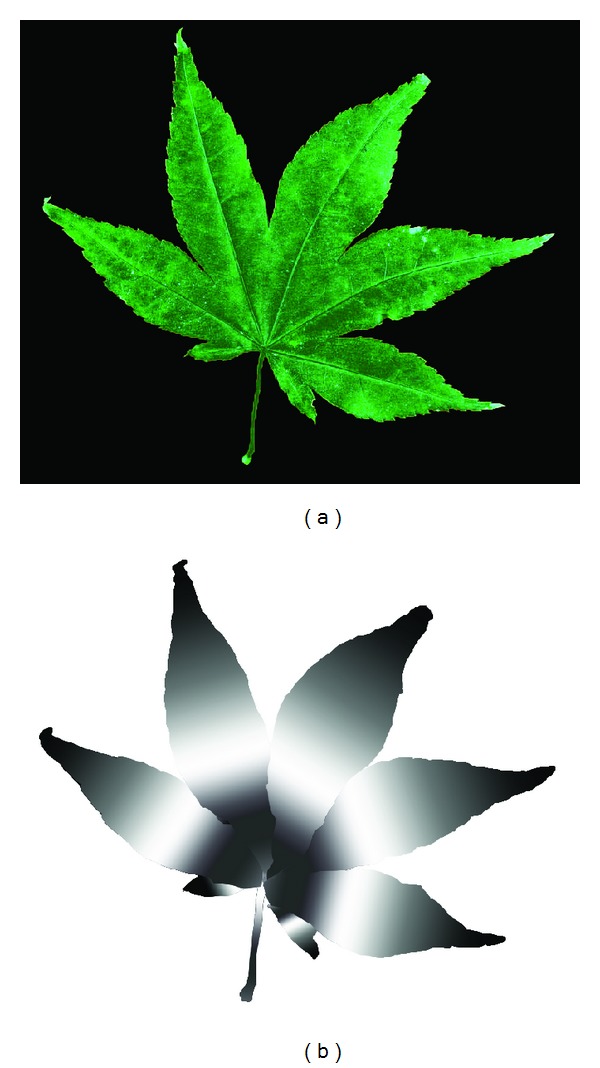
(a) The texture of a maple leaf; (b) mask map of the maple model.

According to Newton's law of motion *F* = *ma*, for the same force *F*, the smaller the object's mass *m* is, the larger the acceleration *a* is. So we can control the deformation of leaves by setting different masses of the particle's. We introduce the *mask map* to adaptively control the particles masses. The *mask map* is generated according to the texture image of the leaf. Suppose that we have a texture image of a leaf called leaf1.bmp which is obtained by scanning the real leaf. We select out the leaf region from the texture and paint different grayscale colors to this region. The intensities of the painted pixels are in proportion to the particle's masses. For example, if we try to set a smaller mass value for a particle, we can paint this pixel in black or an other color close to black. A maple leaf is shown in Figure 3(a). According to our observations of natural maple leaves, the regions around the leaf corner and close to petiole usually undergo more deformation than other regions. So we paint these regions in black or darker gray values while other regions in brighter gray values as shown in Figure 3(b). Different *mask maps* map different masses to the same particles, which results in different deformation results. The corresponding *mask map* needs to be generated based on the natural deformation pattern of the specific leaf.

According to the texture coordinates of the particles of triangular mesh, we find in the mask map the pixels which correspond to particles in the leaf mesh model. The gray values of pixels in the mask map are mapped to the value of particle masses *m* by the following:

(3) $$\begin{array}{c} m=\{\begin{array}{cc} 0.5, & \text{gray}=0\\ \ln\left(\text{gray}+1\right), & \text{gray}!=0 \end{array} \end{array}$$

In (3), the mass value is computed as logarithm of the grayscale value, which makes the change of the masses more gentle and smooth compared with the changes of grayscale values. Such mass distribution is more amenable to yield natural deformation of leaves.

The detailed steps to implement deformation process are shown as follows.

1. Generate the *mask map* to determine the mass distribution of the leaf.
2. Initialize parameter values in our improved mass-spring model. Set the initial velocity and acceleration of particles to be zero. Initialize masses of the particles according to the *mask map*.
3. Establish constraints among particles. The connection between particles (i.e., the mesh topology) determines what other particles directly exert forces on the current particle for the computation of displacements. The constraints are built by three steps as follows.
   
*Step  1*. Find the adjacent triangle faces of current particle. Adjacent faces are those triangles which include a current particle as one of their vertices.
   
*Step  2*. Find the adjacent particles of a current particle. The other two vertices in adjacent triangles are the adjacent particles of a current particle.
   
*Step  3*. Establish the constraints. Set a flag value for each particle to describe whether this particle had been traversed, and initialize the flag value as false. If one particle is traversed, set its flag value as true. Set the constraints between this particle and its adjacent particles if they are not traversed. Thus, all particles are traversed only once, and the constraints are set without duplication. When this particle is moved, the particles having constraints move with it too.
4. Exert the force, and compute the change of position of each particle by numerical calculation in one time step.
5. Repeat the numerical calculation in each time step to obtain the new velocities and accelerations, and update particle positions accordingly to produce deformation effects at different time steps.

**Figure 4:**
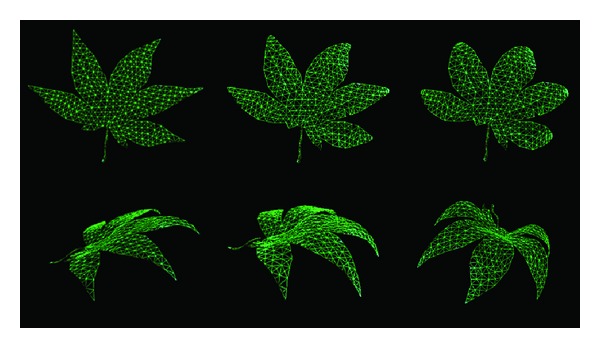
Several deformations using the mask map in Figure 3(b).

For example, the deformation results at different time steps of the maple leaf under the *mask map* in Figure 3(b) are showed in Figure 4 (the first model is the original mesh model).

**Figure 5:**
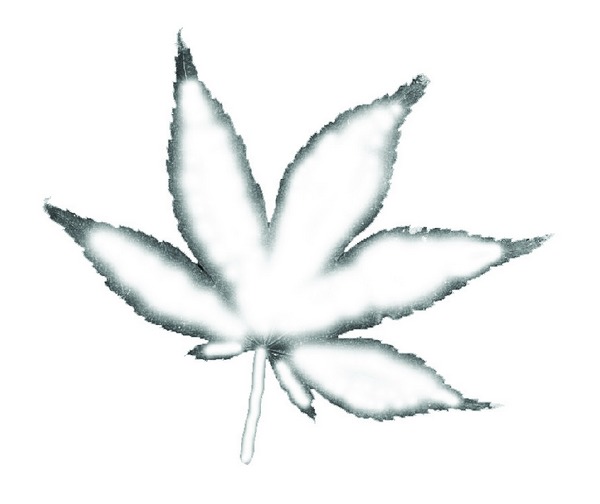
Another mask map of the maple leaf model.

**Figure 6:**
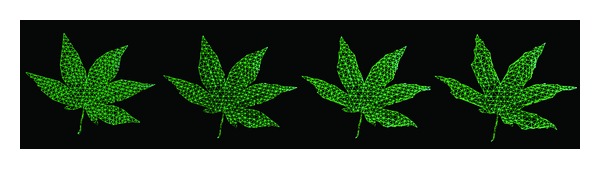
Different deformation results of the maple leaf for mask map shown in Figure 5.

The deformation results in Figure 4 show that the leaf regions with darker gray values are deformed more than the regions with brighter gray values. The masses of those regions with darker gray values are smaller so that they move more distances under forces. The regions with brighter gray values have larger masses which make them move much more slowly. Different movements of particles distributed over the leaf surfaces produce the adaptive deformation results over the leaf surface. If we paint the veins white or bright gray values, we can get the deformation result in which the veins are kept unmoved and two-side regions around veins become curly. With this method, we can control the leave's deformation flexibly. For the same leaf model, we can generate different deformation results by different *mask maps*. In Figure 6, we show the different deformation results for the same leaf model for a different *mask map* in Figure 5. Therefore, in order to achieve desirable deformations, we can construct the corresponding *mask map* to make the leaves deformed as expected.

## 5. Textural and Geometric Changes

To simulate the seasonal changes of leaves, we need to take the transitions of textural colors of leaves into account besides geometric deformations. The whole seasonal changing process of leaves can be regarded as the sequences of a series of discrete states. The leaves transform from one state to the other with certain probabilities conditioned by environmental factors. This transformation can be approximated by Markov chain model [10].

Markov chain model has two properties. (1) The state of the system at time *t* + 1 is only related to the state at time *t* and has nothing to do with the states at a previous time. (2) Transformation of the state from time *t* to time *t* + 1 has nothing to do with the value of *t*. The leaf changing process can be regarded as the Markov chain. Different texture images as well as the deformed geometric shapes are organized to constitute different states in the Markov chain. We simulate various distributions of leaves on the tree by the randomness of the Markov chain model. The environmental factors including temperature and humidness are used as the conditions to determine the probability to transfer from one state to another. By setting different environmental parameters, we get the seasonal appearances of trees with the corresponding distributions of leaves.

**Figure 7:**
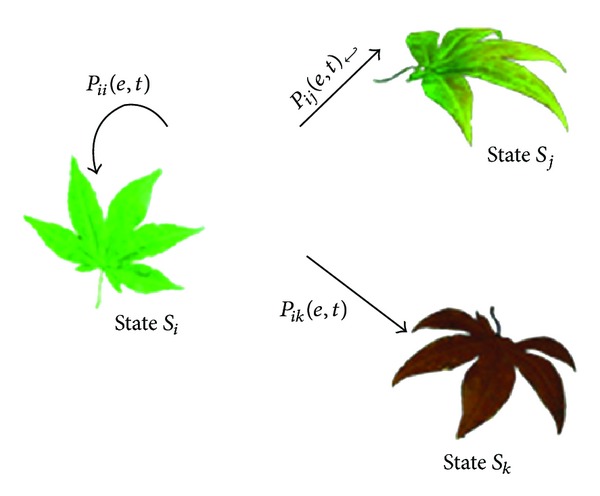
Transition relationship for Markov chain model.

The leaf's state is denoted as *S*_*x*_, where 0 ≤ *x* < *n* and *n* represent the total number of possible states of leaves. Assume that we have three states *S*_*i*_, *S*_*j*_, and *S*_*k*_ and the transition relationship among these three states are shown in Figure 7. It shows that for the state *S*_*i*_ at time *t*, it may evolve to states *S*_*j*_ and *S*_*k*_ or remain in the original state at time *t* + 1 with certain probabilities. 

The arc *P*_*ii*_(*e*, *t*) in Figure 7 represents the possibility that a leaf at a given state *S*_*i*_ stays in the same state at the next time. It is defined as the probability of keeping self-state. The function of this probability is denoted as follows [10]:

(4) $$\begin{array}{c} P_{ii}\left(e,t\right)=e^{-\lambda_{i}(e)t},\;0\leq i\leq n, \end{array}$$

(5) $$\begin{array}{c} \lambda_{i}\left(e\right)=\frac{\ln2}{\tau_{i}\left(e\right)}. \end{array}$$

Function *τ*_*i*_(*e*) is the bilinear interpolation of the temperature and humidness.

The probability that the leaf transfers to other states is denoted as 1 − *P*_*ii*_(*e*, *t*). *P*_*ij*_(*e*, *t*) is defined as the probability of the leaf at state *S*_*i*_ transferring to another state *S*_*j*_, and it is computed by (6) as follows:

(6) $$\begin{array}{c} P_{ij}\left(e,t\right)=\left(1-P_{ii}\left(e,t\right)\right)X_{ij}\left(e\right),\;0\leq i≺n,\;\;i\neq j. \end{array}$$

Function *X*_*ij*_(*e*) is the bilinear interpolation of four constants between zero and one. These four constants correspond to the transition possibilities in the four extreme cases: wet and cold, wet and warm, dry and cold, and dry and warm. The values of these constants are interactively specified by users. 

**Figure 8:**
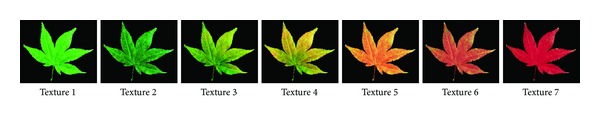
Seven texture states of a maple model.

The parameters of time, temperature, and humidness are set by users. Taking the maple leaves in Figure 8, for example, we use three specific combinations of textures and shapes for each season. For instance, three main states are used to represent leaves in summer, which are texture 2 in Figure 8 combined with the first deformation in Figure 4, texture 3 combined with the second deformation, and texture 4 combined with the third deformation.

**Figure 9:**
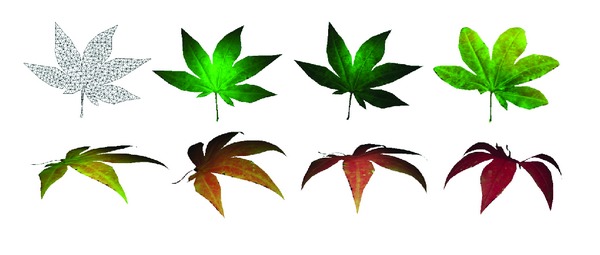
The basic triangular mesh model of the maple leaf, and seven states combining textures and geometric deformations.

Several states which combine changes of textures and shapes in different seasons are showed in Figure 9. Given the combinations of states, we calculate the transition probabilities of leaves according to the specific temperature and humidness set for certain seasons and get the corresponding leave's distributions in that season.

**Figure 10:**
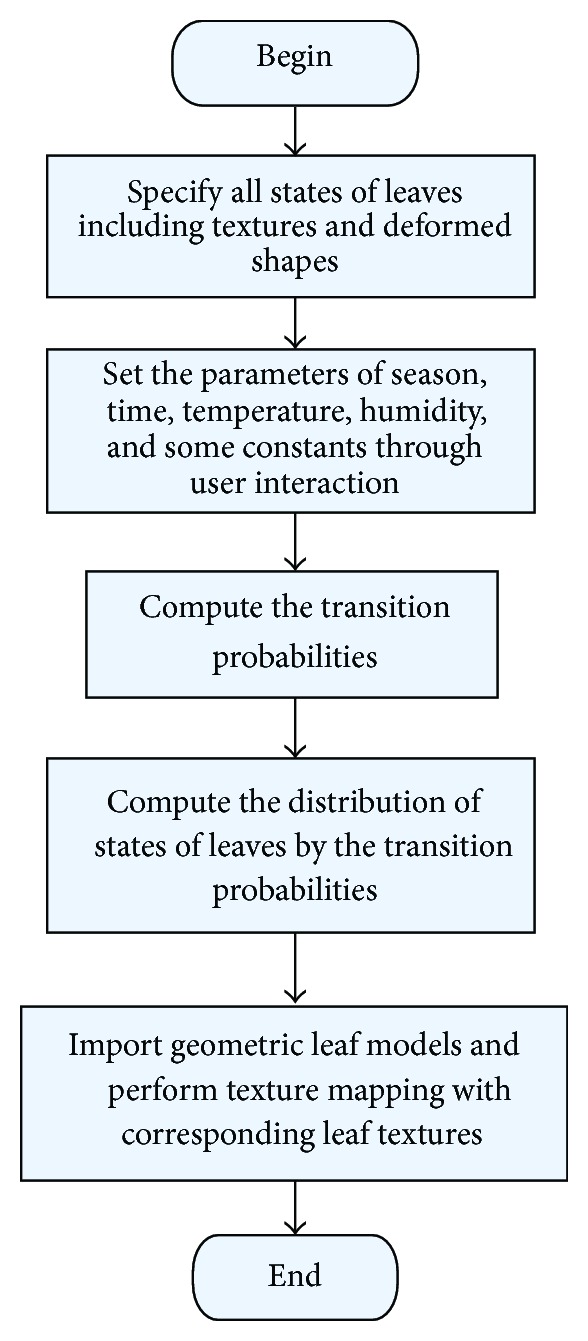
Seasonal changing process of leaves based on Markov-chain model.

To summarize, the seasonal changing process of leaves under certain environmental parameters is showed in Figure 10.

## 6. Results

**Figure 11:**
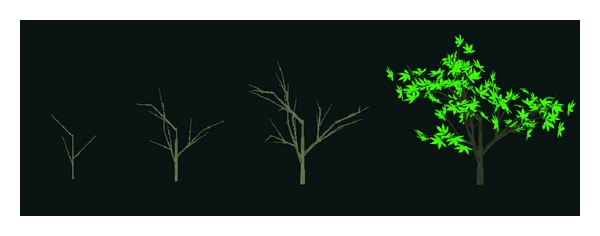
Tree growing process based on L-system.

**Figure 12:**
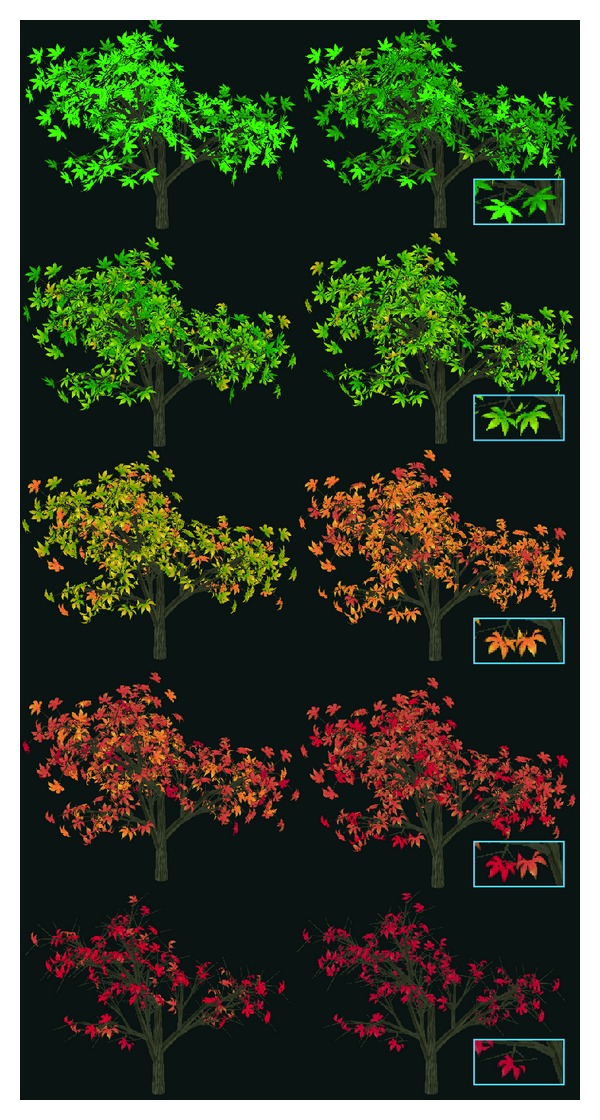
Seasonal changes of a maple tree based on Markov chain model.

To produce the results of seasonal changes of trees, we grow the leaves on the trees and simulate their distributions for different seasons. In order to get the 3D model of the tree, we adopt the L-system method to produce the trunks and branches of the tree. The trunks and branches of the tree are drawn with quadratic surface, and the leaves grown on branches are modeled as triangular meshes. In Figure 11, we model the tree and its growth through the iteration of the L-system, and the leaves grown on the tree are shown. To simulate leaves, seasonal changes, we distribute various leaves on the tree under different environments based on Markov chain model.  Figure 12 shows some seasonal changes of the maple tree, and the enlarged picture at the lower right corner show the change of the individual leaf more clearly.

## 7. Conclusion

In this paper, we propose a computational approach to simulate the seasonal changes of living leaves by combining the changes in geometric shapes and textural colors. First, the key points are selected on the leaf image by user interaction. Then, the triangular mesh of the leaf is constructed and optimized by improved Delaunay triangulation. After the models of leaves have been obtained, the deformations of leaves are computed by improved mass-spring models. The seasonal changes of trees under different environmental parameters are computed based on Markov chain. The improved mass-spring model is based on the user-specified *mask map* which adaptively determines the masses of particles on the leaf surface. 

In the future, we are interested in the following work.

1. Work on how to generate the mask map more naturally according to the characteristics of the deformations of leaves.
2. Intend to simulate the dynamic procedure of the leaves falling onto ground out of gravity.
3. Develop a more precise model to compute the colors of leaves which takes into account of the semitransparency of leaves.
